# Role of Distinct Natural Killer Cell Subsets in Anticancer Response

**DOI:** 10.3389/fimmu.2017.00293

**Published:** 2017-03-16

**Authors:** Helena Stabile, Cinzia Fionda, Angela Gismondi, Angela Santoni

**Affiliations:** ^1^Department of Molecular Medicine, Sapienza University of Rome, Rome, Italy; ^2^Italian Institute of Technology, Sapienza University of Rome, Rome, Italy; ^3^Istituto Pasteur-Fondazione Cenci Bolognetti, Sapienza University of Rome, Rome, Italy

**Keywords:** natural killer cell subset, tumor microenvironment, natural killer cells, hematological malignancies, solid tumors

## Abstract

Natural killer (NK) cells, the prototypic member of innate lymphoid cells, are important effectors of anticancer immune response. These cells can survey and control tumor initiation due to their capability to recognize and kill malignant cells and to regulate the adaptive immune response *via* cytokines and chemokines release. However, several studies have shown that tumor-infiltrating NK cells associated with advanced disease can have profound functional defects and display protumor activity. This evidence indicates that NK cell behavior undergoes crucial alterations during cancer progression. Moreover, a further level of complexity is due to the extensive heterogeneity and plasticity of these lymphocytes, implying that different NK cell subsets, endowed with specific phenotypic and functional features, may be involved and play distinct roles in the tumor context. Accordingly, many studies reported the enrichment of selective NK cell subsets within tumor tissue, whereas the underlying mechanisms are not fully elucidated. A malignant microenvironment can significantly impact NK cell activity, by recruiting specific subpopulations and/or influencing their developmental programming or the acquisition of a mature phenotype; in particular, neoplastic, stroma and immune cells, or tumor-derived factors take part in these processes. In this review, we will summarize and discuss the recently acquired knowledge on the possible contribution of distinct NK cell subsets in the control and/or progression of solid and hematological malignancies. Moreover, we will address emerging evidence regarding the role of different components of tumor microenvironment on shaping NK cell response.

## Introduction

Natural killer (NK) cells are innate lymphoid cells (ILCs) (1) with a crucial role in immunosurveillance. They display cytotoxic activities against transformed or viral infected cells but are also an important source of chemokines and cytokines highly impacting on adaptive immune responses (2, 3).

Natural killer cell activity is dependent on activating and inhibitory signals transmitted by a large repertoire of surface receptors. Inhibitory receptors prevent NK cells from killing healthy cells and include KIRs, CD94/NKG2A, and ILT2/CD85. The activating receptors recognize self-proteins mainly expressed on stressed target cells and include NCRs (NKp46, NKp30, NKp44), NKG2D, and DNAM1, among others (4).

Natural killer cells develop in the bone marrow (BM) from lineage restricted progenitors, although maturation can also occur in the periphery (5–7). Fully mature NK cells circulate in the peripheral blood (PB), where they represent 5–20% of total lymphocytes, but they are also found in several lymphoid and non-lymphoid organs (8, 9).

Phenotypically, NK cells are defined by the expression of CD56 and the lack of CD3–TCR complex. Moreover, based on CD16 and CD56 expression levels, they are classically distinguished in two subsets: CD56^high^CD16^±^ and CD56^low^CD16^high^. The CD56^low^CD16^high^ NK cell subset expresses high levels of KIRs, the maturation marker CD57, and mediates natural and antibody-dependent cellular cytotoxicity, exhibiting high levels of perforin and enhanced killing; CD56^high^CD16^±^ NK cells are characterized by NKG2A, low levels of perforin, and are primarily specialized for cytokine production. It is still debated whether these subsets are functionally distinct NK cells or different stages of maturation. A linear differentiation relationship between CD56^high^ CD16^±^ NK cells and CD56^low^CD16^high^ NK cells has been proposed (10, 11), but it is not supported by observations on human NK cell deficiencies (12, 13); moreover, the possibility that tissue-resident NK cells develop locally is also considered (14).

Besides CD56^high^CD16^±^ and CD56^low^CD16^high^, additional NK cell subpopulations have been identified under normal and pathological conditions, based on their receptor repertoire (15–17). Thus, human NK cells emerge as a highly heterogeneous and plastic population including subtypes with different and specific functions.

Natural killer cell subsets also differ in tissue distribution that is related to distinct homing properties and/or *in situ* maturation. Tissue-resident NK cells express a different pattern of chemokine and adhesion receptors and also differ from their blood-circulating counterpart (18, 19). PB CD56^high^CD16^±^ NK cells express CD62L, CCR7, CXCR4, and CXCR3 that allow their preferential recruitment to secondary lymphoid organs, tumor, and inflamed tissues (8, 20, 21). Conversely, resident CD56^high^ NK cells lack CD62L but express other adhesion molecules, including the α integrin subunit CD49a and CD103 (22). The CD56^low^CD16^high^ NK cell subset expresses low level of CD62L and lacks CCR7, but it is characterized by CXCR4, CX_3_CR1, CXCR2, and CXCR3 chemokine receptors responsible for their migration into the inflamed sites.

Natural killer cells play a major role in tumor immunosurveillance. They can control tumor initiation but are often inefficacious in advanced disease. More recently, strong NK cell infiltration in established cancers also suggested a role in disease progression (23, 24). Tumor-infiltrating NK cells (TINKs) share phenotypic and functional properties with decidual NK cells (dNKs), well known for their regulatory, pro-angiogenic, and low cytotoxic activities (23, 25, 26).

In tumor microenvironment, several cellular and soluble factors affect NK cell phenotype and function and promote tumor cell evasion from NK cell-mediated recognition and killing (27).

Because the capability of distinct NK cell subsets to exert specific functions, it is extremely important to understand which subpopulations mediate the antitumor response and which environmental factors modulate their activity. Here, we review the role of distinct NK cell subsets in human solid and hematological cancers and the impact of tumor microenvironment on their phenotypic and functional features.

## NK Cell Subsets in Solid Tumors

Neoplastic transformation was shown to significantly alter NK cell subset localization (Table 1), though the exact role of the TINKs subsets remains poorly characterized (28, 29).

**Table 1:** **Phenotype of NK cell subsets in tumors**.

Natural killer (NK) cell subset		Tumor	Phenotype	Function		Reference
				Cytokine production	Cytotoxicity	
Solid tumors	CD56^high^perforin^low^	Lung and breast cancer	NKG2A^+^CD27^+^KIR^+^ CD62L downregulation	ND	ND	(20)
	CD56^high^CD16^low^	Breast, melanoma, and colon cancer	CD9^+^, CXCR3^+^	VEGF	ND	(25)
	CD56^high^CD16^−^	Non-small cell lung cancer	KIR^+^CD69^+^HLA-DR^+^ NKp44 upregulation	High production of VEGF, PLGF, IL-8	No cytotoxicity	(26)
	CD56^high^	Prostate	NKp46, NKG2D, NKp30, DNAM1, CD16 downregulation ILT2 upregulation	ND	No cytotoxicity	(30)
	CD56^hight^CD16^+^	Metastatic lymph nodes adjacent to metastatic melanoma	NKp46^+^, NKG2D^+^, NKp30^+^, CD158 (a, b and e)^+^	ND	Low cytotoxicity	(31)
	CD56^low^	Metastatic lymph nodes from melanoma patients	KIR^+^CD57^+^CD69^+^CCR7^+^	ND	High cytotoxicity	(32, 33)
	CD56^low^	Non-small cell lung cancer	NKp46^+^, NKp80, CD16, NKG2D, and DNAM-1 downregulation	No IFN-γ production	No cytotoxicity	(34)
	CD56hight	Intestinal stromal cancer	CD16^−^KIR^−^NKp30c^+^	Reduced production of TNF-α IFN-γ production	Reduced cytotoxicity	(35)
Hematologic tumors	CD56NCR^dull^	AML	CD16^+^KIR^+^	ND	No cytotoxicity	(36)
	CD56NCR^high^	AML	ND	ND	No cytotoxicity	(36)
	CD56NKp46^low^	AML/B-ALL	NKG2A upregulation	No IFN-γ production	No cytotoxicity	(37)
	CD56^low^CD16^low^	B-ALL/T-ALL	ND	No IFN-γ production	No cytotoxicity	(38)
	CD56^+^	Myelodysplastic syndromes (MDS)	NKG2D (PB/BM) DNAM1 (BM) downregulation	ND	No cytotoxicity	(39)
	CD56^low^	Multiple myeloma (MM)	DNAM1, CD16, 2B4 downregulation	ND	No cytotoxicity	(40, 41)
	CD56	MM	CD161 downregulation and CD158a upregulation	ND	No cytotoxicity	(40)
	CD56^low^	AML	KIR^+^CD57^+^	ND	ND	(42)
	CD56^low^	AML	CD16/CD57^high^	ND	ND	(43)
	CD56^low^	MDS	KIR^−^NKG2A^−^	ND	ND	(44)

*up/downregulation, reduction are determined with respect to NK cells from healthy individuals; ND, not determined*.

The study of TINKs in solid tumors is rather complex as phenotypic alterations can occur following isolation and a comparison with the healthy tissue counterpart is difficult to perform.

Like tissue-resident NK cells that are generally CD56^high^CD16^low^ and more specialized for cytokine production, a prevalence of CD56^high^ NK cells can infiltrate solid malignancies, although they can exhibit features and/or functions other than those of their circulating and/or healthy counterpart tissue. Thus, a significantly higher frequency of CD56^high^perforin^low^ NK cells was observed in breast and lung cancers, with respect to normal tissues. CD56^high^ NK cells were poorly cytotoxic, but cytokine producers, and were mainly localized within the stromal compartment. CD56^high^perforin^low^ accumulation was not attributed to major tumor microenvironment-driven NK cell developmental alterations, but rather to a peculiar chemokine milieu. Indeed, downregulation of CXCL2 that specifically attracts CD56^low^ NK cells and upregulation of CXCL9 and CXCL10 that specifically support CD56^high^ NK cells homing were observed (20, 45).

In breast cancer patients, five different circulating NK subsets were also identified (46): CD56^low^CD16^+^, CD56^low^CD16^−^, CD56^high^CD16^−^, CD56^high^CD16^+^, and CD56^−^CD16^+^. A higher percentage of CD56^low^CD16^−^ and CD56^high^CD16^−^ subsets were observed both in PB and in advanced invasive mammary tumors. Furthermore, by phenotypic and functional analysis, both subpopulations emerged as more immature (CD117^high^CD27^high^CD57^low^) and less functional (low levels of activating receptors, perforin, and granzyme B and degranulation capability). Collectively, these observations suggest that breast tumor microenvironment blocks or reverses NK cell maturation, favoring the emergence of non-cytotoxic NK cells.

Changes in the expression patterns of activating and inhibitory receptors have been also described in tumor-associated CD56^high^ NK cells and have been implicated in their functional deficits. CD56^high^ NK cells, displaying an immature and activated phenotype associated with low or null degranulation potential, were found in prostate tumor and area selected out of the tumor site (30). However, in prostate cancer, lower expression of some activating receptors (NKp46, NKp30, NKG2D, DNAM-1, CD16) and higher expression of the inhibitory receptor ILT2 were observed, with more pronounced effects in NK cells infiltrating metastatic than localized tumors; these latter data indicate that tumor microenvironment can impair NK cytotoxic functions by altering the balance between NK activating and inhibitory receptors. The analysis of NK cell subsets in the lymph nodes of cancer patients revealed comparable numbers of CD56^high^ NK cells in the regional metastatic lymph nodes from stage III melanoma patients (M-LN) and mediastinal lymph nodes from healthy donors (HD). However, 40–60% of CD56^high^ NK cells in M-LN also expressed CD16. CD56^high^CD16^+^ NK cells displayed an activated phenotype, and their *ex vivo* degranulating capacity inversely correlated with the percentage of malignant cells, suggesting a local tumor-induced suppression of NK cell activation. The prevalence of CD56^high^CD16^+^ NK cells in M-LNs was attributed to the maturation and activation of tumor resident CD56^high^CD16^−^ NK cells and/or to the migration of PB CD56^+^CD62L^+^ NK cells to M-LNs, where CD16 expression could be upregulated (31).

A relevant property of CD56^high^CD16^−^ NK cells within different solid tumors, such as breast, melanoma, colon cancer (25), non-small lung cancer (26) is their pro-angiogenic phenotype possibly responsible for their tumor-promoting role. Indeed, unlike circulating CD56^high^CD16^−^ but similar to dNK cells, CD56^high^CD16^−^ TINK cells express high levels of CD9, CXCR3, produce VEGF, and have a lower cytotoxic potential, suggesting that similar maturation/polarization mechanisms occur in the decidua and tumor microenvironment of PB NK cells (47–49).

Although substantial evidence indicates CD56^high^CD16^low^ NK cells as the major TINK, there are also reports on tumor infiltration by CD56^low^ NK cells (32, 33, 46). Enrichment in the tumor infiltrated lymph nodes (TILN) and concomitant reduction of CD56^low^ NK cells in PB were observed in melanoma patients. These CD56^low^ (CD57^+^CD69^+^CCR7^+^KIR^+^) NK cells were highly cytotoxic against autologous melanoma cells, and, in accordance with their homing into TILN, they expressed CCR7. The reduced proportion of CD56^low^ NK cells in the PB supports the possibility of a selective recruitment of this subset in TILN. However, *in situ* maturation of CD56^high^ NK into more cytotoxic CD56^low^ NK cells was also suggested because the different chemokine milieu dominated by CXCL8 and CCL2, which may recruit both CD56^high^ and CD56^dim^ CXCR2^+^/CCR2^+^ PB NK cells into the TILN (32).

The emerging concept of tissue-specific functions of NK cells together with the selective enrichment of specific subsets in neoplastic tissues indicate that the outcome of antitumor NK cell effector functions is not always predictable and largely depends on the particular tumor microenvironment.

## NK Cell Subsets in Hematological Malignancies

A large body of evidence indicates that NK cells play a preferential role in the control of the onset and progression of hematological tumors. Moreover, unlike solid cancers where monitoring of PB NK cells could not provide correct information on their tumor-infiltrating counterpart, evaluation of circulating NK cell status can be highly relevant in the context of hematological malignancies.

Abnormal NK cell cytolytic function was observed in acute and chronic leukemia (AML-ALL and CLL-CML), myelodysplastic syndromes (MDS), and multiple myeloma (MM). Yet, most of the studies are focused on PB, but not BM, and poorly address NK cell phenotypic and functional heterogeneity.

The main receptors involved in NK cell recognition and killing of leukemic blasts are NCRs, NKG2D, and DNAM1. According to NCR surface density, unlike NK cells from HD that are mainly NCR^high^ (50), a NCR^low^CD16^+^KIR^+^ NK cell subset that failed to recognize and kill autologous and allogeneic blasts was described in AML patients (36). A smaller cohort of AML patients was also characterized by the presence of the NCR^high^ NK cell subset that showed impaired cytotoxic activity, probably due to NCR ligand down-modulation on leukemic cells. In addition, significant reduction of NKp46 together with increased NKG2A expression was associated with functionally impaired PB NK cells from AML patients with respect to HD (37). Similar to AML, the frequency of PB NCR^+^ and in particular NKp46^+^ NK cells from B-ALL patients was lower. Moreover, they also displayed increased NKG2A expression. These phenotypic abnormalities were associated with impaired NK cell killer ability and IFN-γ production in response to autologous blasts (51). As regards to other activating NK receptors, a lower frequency of NKG2D^+^ and DNAM-1^+^ NK cells was observed in the context of MDS, AML, and MM (39, 52); moreover, NK cells from MM patients also displayed reduced levels of CD244, CD16, and CD161 (40, 41, 53, 54).

A different scenario was observed with CLL and CML CD56^low^ NK cells which exhibited the same profile of activating and inhibitory receptors of HD but reduced NK cytotoxic ability (55).

Recently, we reported an increased frequency of a newly identified NK cell subset characterized by low levels of CD56 and CD16 (CD56^low^CD16^low^) and NKG2A^+^ in both BM and PB of pediatric B-ALL and T-ALL. In HD, this subset was endowed with both higher cytotoxic activity and IFN-γ producing ability, but it resulted functionally impaired in leukemic patients (38). Similarly, a higher frequency of non-cytotoxic CD56^low^CD16^low^ NK cells was found in advanced breast cancer (46), suggesting both a preferential homing and functional alterations of this subset in tumor-microenvironment.

Overall, these findings suggest that several mechanisms, including downregulation of activating receptors and/or upregulation of inhibitory receptors on NK cells or modulation of their ligands on cancer cells are responsible for tumor escape from NK cell recognition in hematological malignancies.

In the context of hematological cancers where tumor cells are present in the BM that represents the main site of NK cell differentiation, an important question to address is whether tumor growth also affects NK cell development. Most of the studies, however, addressed this issue examining PB and not BM NK cells. In this regard, Chretien et al. (42) compared the presence of five different stages of NK cell development (CD56^high^, CD56^low^/KIR^−^/CD57^−^, CD56^low^/KIR^+^/CD57^−^, CD56^low^/KIR^−^/CD57^+^, and CD56^low^/KIR^+^/CD57^+^) in the PB of AML patients and found that one-third of the patients exhibited a significant increase in the proportion of the more mature CD56^low^/KIR^+^/CD57^+^ NK cells at the expenses of more immature CD56^high^ NK cell subset. In addition, a recent study on NK cells from the BM of AML patients showed a reduced frequency of the more mature CD56^low^CD16/57^high^ NK cell subset that did not correlate with a good prognosis (43). Collectively, these findings, although suggestive of a possible influence of AML cells on NK cell development, are still incomplete, as BM and PB NK cell subsets from the same patient have not been examined, and the possibility that the observed phenotype is due to a preferential migration of more mature CD56^low^/KIR^+^/CD57^+^ NK cells from BM to PB is still open (56).

Moreover, it is increasingly understood the impact of hematological tumors on BM stromal cells, which are crucial for an optimal NK cell differentiation. In this regard, evidences on altered chemokine and cytokine production by BM stromal cells were provided (44), suggesting that effects on NK cell differentiation can be due to the lack of a proper stromal support for NK cell progenitors and/or an altered NK cell subset trafficking.

## NK Cell Subsets and Tumor Microenvironment

Several studies indicate that tumor-induced impairment of NK cell functions correlates with alterations of NK cell subset distribution. On the other hand, different immunosuppressive mechanisms can be also responsible for functional NK cell impairment in solid and hematologic malignancies.

**Figure 1:**
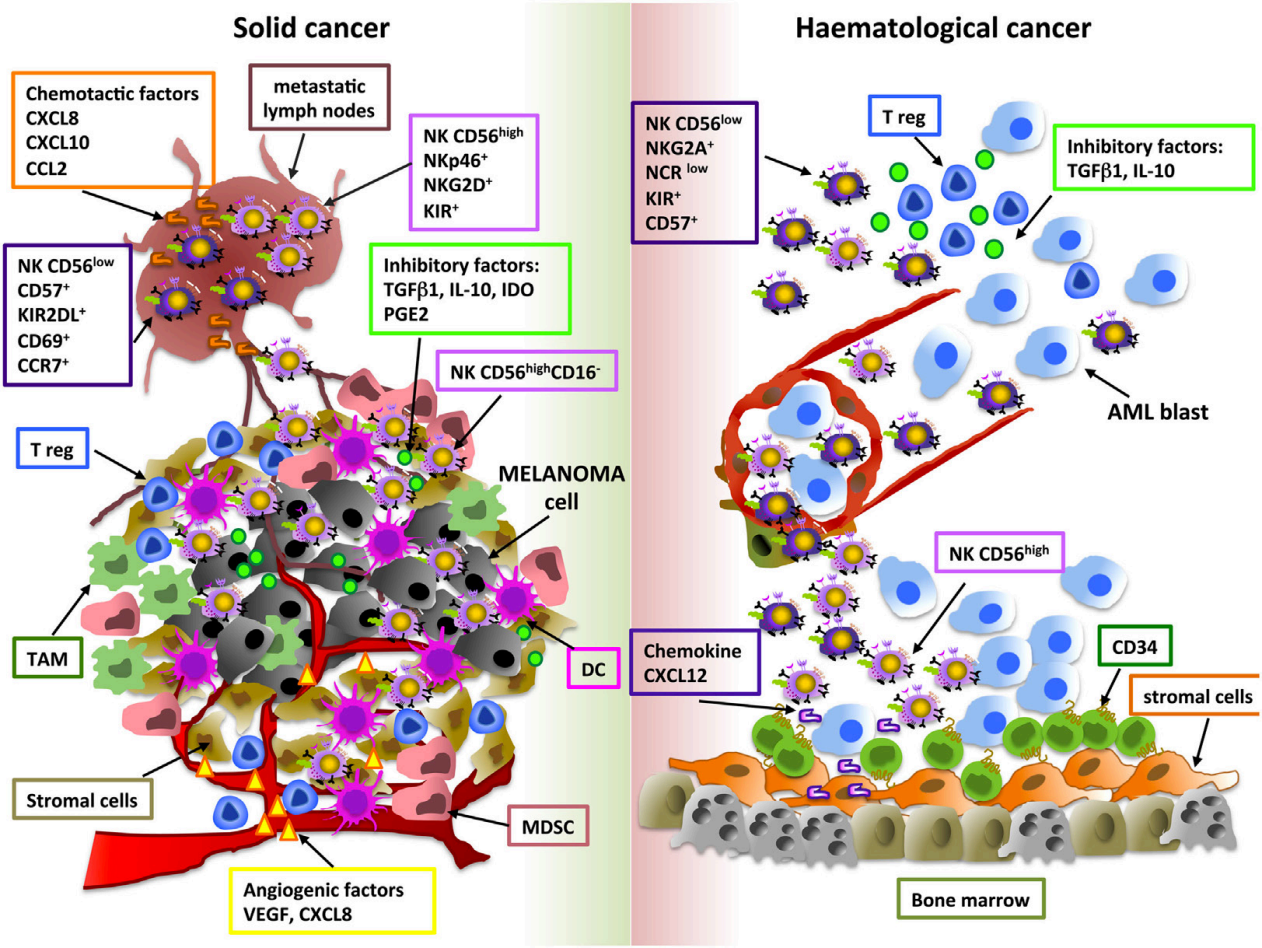
**Shaping natural killer (NK) cell subsets in tumor microenvironment**. The main cellular and soluble factors affecting NK cell subset distribution in tumor microenvironment are shown. Melanoma and AML are reported as examples of solid and hematological tumor, respectively. For melanoma, tumor and metastatic lymph node (M-LN) infiltrating NK cell subsets are represented. For AML, bone marrow and peripheral blood NK cell subpopulations are indicated.

Tumor-related soluble factors may be responsible for phenotypic and functional alterations of NK cells, moreover different tumor-resident immune cells, such as M2-polarized macrophages, MDSC, DC, and Treg, may affect NK cell activity, by releasing soluble factors (e.g., IL-10, IDO, PGE_2_) or by direct contact-dependent mechanisms (57–59) (Figure 1). Although higher amount of TGF-β1, PGE2, IL-10, and IDO were detected in supernatants of solid and hematological tumors, the impact of these soluble factors on NK cell subset distribution was suggested only based on *in vitro* observations (30, 51). Differently, Mamessier et al. performed *in vivo* correlation studies demonstrating that in breast cancer patients decreased expression of activating NK cell receptors (NKp30, NKp46, NKG2D, DNAM-1) or cytotoxic molecules (GZMB) and increased levels of the inhibitory receptor NKG2A on NK cells were associated with high amount of TGF-β1 and PGE2 in tumor supernatants. In particular, TGF-β1 and PGE2 were shown to negatively correlate with molecules related to NK cell cytoxicity and positively correlate with NKG2A receptor expression (60), thus suggesting that these molecules play a role in these regulatory mechanisms.

Among these factors, particular attention has been given to TGF-β1, which has been shown to exert several effects on NK cells, including inhibition of proliferation and *in vitro* NK cell development and differentiation. In this regard, this cytokine was found to reduce the number of NK cells developing from human CD34^+^ progenitor cells and to promote the conversion of PB CD56^high^CD16^+^ NK cells into a dNK-like CD56^high^CD16^−^ phenotype (61, 62). Thus, also at tumor site, TGF-β1 may take into account of the pro-angiogenic dNK-like phenotype of tumor-infiltrating NK cells. A number of studies suggest that tumor-derived TGF-β1 also impacts NK development in the context of hematological malignancies. In particular, TGF-β1 overexpression in the BM tumor-microenvironment (MDS, CML, and MM) may be responsible for the suppressive effect of cancer cells on BM stromal cells, thus compromising their supportive role on NK cell maturation (44, 63, 64). Finally, this cytokine may also interfere with intra-tumoral NK cell infiltration *via* modulation of their chemokine receptors (65). In this regard, a peculiar chemokine milieu has been proposed to be important for the recruitment of specific NK cell subpopulations in a number of solid tumors; moreover, altered chemokine expression patterns may also affect NK cell trafficking in hematological malignancies (32, 44, 45). However, higher concentration of chemokines does not always correlate with the presence of these lymphocytes in tumor microenvironment, thus suggesting that other and more complex mechanisms can affect their recruitment (66).

## Tumor Escape from NK Cell-Mediated Recognition and Killing

Elusion of NK cell recognition is a major mechanism of tumor immune evasion. NK cell-activating ligands are expressed on malignant cells, but they can be also released in a soluble form through metalloproteinase-mediated cleavage, exosome secretion, or alternative splicing. Indeed, soluble forms of these ligands are present in the serum or peritoneal fluids of various cancer patients, and their levels positively correlate with tumor stage, metastasis, and poor prognosis (67–69). Reduction of activating ligand expression on cancer cells leads to a less efficient recognition and killing by cytotoxic lymphocytes. Concomitantly, soluble ligands can engage their receptors and cause their internalization in NK cells; accordingly, a negative correlation between soluble ligands and NKG2D expression on NK cells was largely documented in both solid and hematological tumors (70, 71). However, conflicting results have described either inhibition or promotion of NK cell activation following NKG2D endocytosis (72). An additional escape strategy used by cancer cells is based on the dominance of NK cell inhibitory signals. In several cancer cells, expression of MHC class I molecules binding to inhibitory KIR receptors (KIR2DL2/3, KIR3DL1, and KIR2DL1) results in switch off NK cell effector functions (37, 73–75). Moreover, high levels of non-classical antigens HLA-G (ligand of ILT-2 and KIRDL-4) and HLA-E (ligand of NKG2A/CD94) were found in tumor and serum of cancer patients and were considered independent markers of poor prognosis in various malignancies (76–79). Finally, tumor cell overexpression of other ligands triggering inhibitory signals on NK cells, such as PDL-1/2, contributes to inhibit their susceptibility to NK cell-mediated killing (80, 81).

## Conclusion

Accumulating evidence indicates that, far from the simple and first distinction in two subsets, NK cells are a very highly heterogeneous population, and different marker combinations can be used to identify distinct subpopulations endowed with specific functional properties. Based on these observations, the role of different NK cell subsets in pathological contexts, including cancer, is increasingly elucidated. Moreover, the emerging evidence about different ILC populations further raise the necessity of a more detailed molecular phenotypic and functional characterization of innate lymphoid subsets in the cancer context. The identification of the role played by the different NK cells both in solid and hematological malignancies would be valuable for the design of novel NK cell targeted therapeutic interventions.

## Author Contributions

HS, CF, AG, and AS contributed equally to writing and critically revised the paper.

## Conflict of Interest Statement

The authors declare that the research was conducted in the absence of any commercial or financial relationships that could be construed as a potential conflict of interest.
